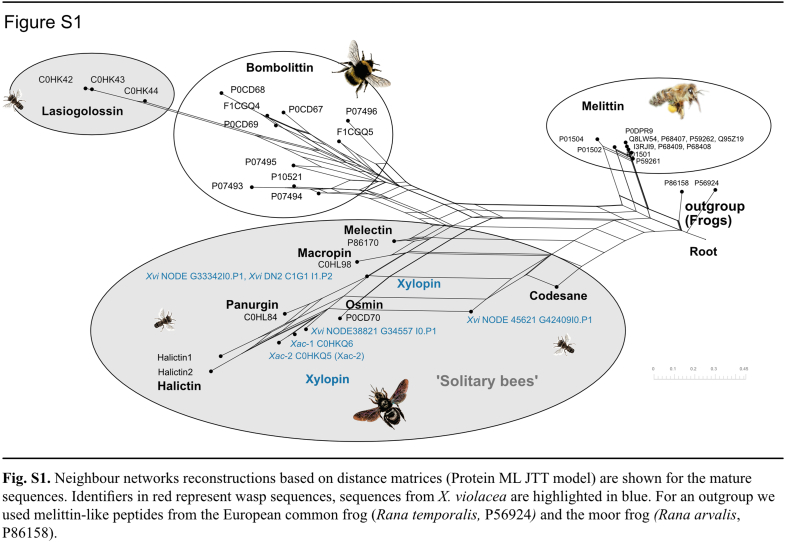# Venom profile of the European carpenter bee *Xylocopa violacea*: Evolutionary and applied considerations on its toxin components

**DOI:** 10.1016/j.toxcx.2022.100117

**Published:** 2022-03-10

**Authors:** Björn M. von Reumont, Sebastien Dutertre, Ivan Koludarov

**Affiliations:** a Goethe University Frankfurt, Institute for Cell Biology and Neuroscience, Department for Applied Bioinformatics, 60438, Frankfurt am Main, Germany; b Justus Liebig University of Giessen, Institute for Insect Biotechnology, Heinrich-Buff-Ring 58, 35392, Giessen, Germany; c LOEWE Centre for Translational Biodiversity Genomics (LOEWE TBG), Senckenberganlage 25, 60325, Frankfurt, Germany; d IBMM, Université Montpellier, CNRS, ENSCM, 34095, Montpellier, France

**Keywords:** Bee venom, Xylopin, Solitary bees, Melittin, Apamin, Proteo-transcriptomics

## Abstract

Modern venomics is increasing its focus on hymenopterans such as honeybees, bumblebees, parasitoid wasps, ants and true wasps. However solitary bees remain understudied in comparison and the few available venom studies focus on short melittin-like sequences and antimicrobial peptides. Herein we describe the first comprehensive venom profile of a solitary bee, the violet carpenter bee *Xylocopa violacea*, by using proteo-transcriptomics. We reveal a diverse and complex venom profile with 43 different protein families identified from dissected venom gland extracts of which 32 are also detected in the defensively injected venom. Melittin and apamin are the most highly secreted components, followed by Phospholipase A2, Icarapin, Secapin and three novel components. Other components, including eight novel protein families, are rather lowly expressed. We further identify multiple forms of apamin-like peptides. The melittin-like sequences of solitary bees separate into two clades, one comprised most sequences from solitary bees including xylopin (the variant in *Xylocopa*), while sequences from *Lasioglossa* appear closer related to melittin-like peptides from *Bombus* (Bombolittins). Our study suggests that more proteo-transcriptomic data from other solitary bees should be complemented with corresponding genome data to fully understand the evolution and complexity of bee venom proteins, and is of a particular need to disentangle the ambiguous phylogenetic relations of short peptides.

## Highlights

• Venoms of solitary bees are so far surprisingly little studied.
• We analyse here the venom profile from the solitary carpenter bee *X. violacea*.
• The venom of *X. violacea* is similar complex as in honey bees.
• Besides new melittin variants eight novel venom components are identified.
• Our results contribute to understand better the evoluiton of bee venoms.

## Graphical abstract

**Figure:**
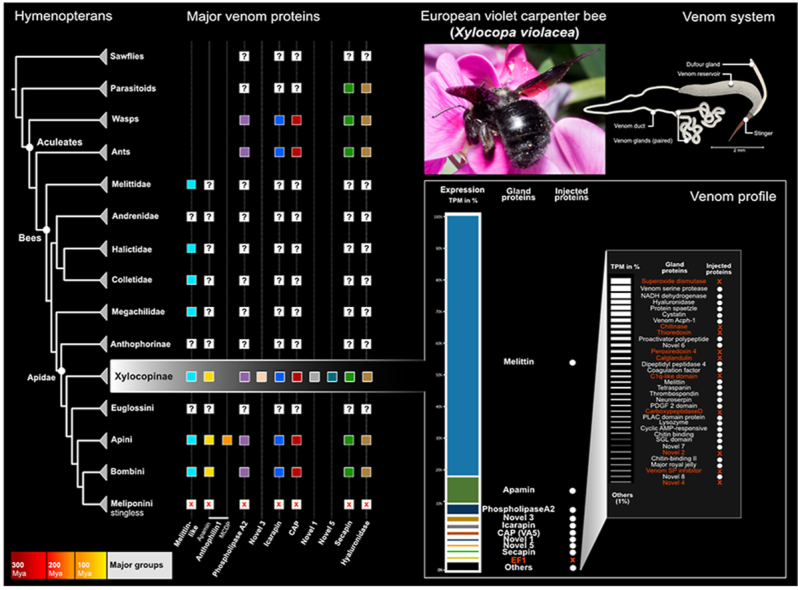

## 1 Introduction

**Fig. 1:**
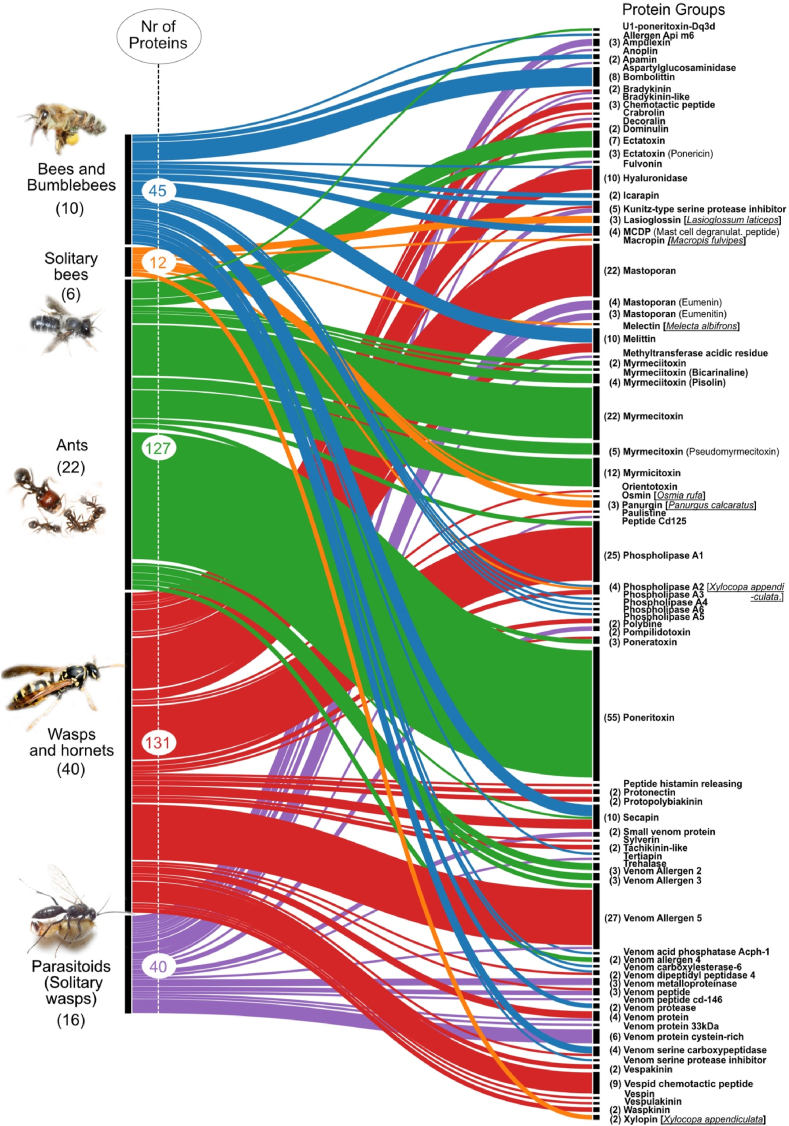
Alluvial chart illustrating the 355 curated hymenopteran venom proteins and peptides. Data was mined in Uniprot using the keywords #Hymenotpera and #venom (December 2021). The number of species per group are given in brackets, the groups are colour-coded (blue = bees and bumblebees, orange = solitary bees, green = ants, red = wasps and hornets, purple = parasitoids and solitary wasps). On the right the protein groups are shown, the number of sequences per group are indicated in brackets, for unique single proteins no number is given. Please note that sequences from melittin-like codesan (from *Colletes daviesanus*) and halictin (from *Halictus sexcinctus*) are not included here. Their sequences are only available as text in the respective manuscripts and are not provided in databases. The species names of solitary bees are underlined and given in brackets.

Hymenopteran insects are the most species-rich animal group on earth including over one million estimated species (Oeyen et al., 2020; Peters et al., 2017). One of their most iconic evolutionary adaptation is the use of venom, which they employ predominately for defensive and predatory purposes (Kukuk et al., 1989; Piek, 1986; Schmidt, 1982; Schmidt et al., 1986). Scientific reports and observations on eusocial and solitary aculeate hymenopterans (distinct by their characteristic waist and the very maneuverable stinger) have been made since the 17th century and include original studies on their venom system and venom properties (Piek, 1986). These early venom studies focused mostly on species that live in close proximity to humans, in particular honeybees, eusocial wasps as well as parasitoid wasps (Leluk et al., 1989; Piek, 1986; Schmidt et al., 1986). Recently, proteomic, transcriptomic and proteo-transcriptomic analyses have been published that describe in greater detail venom compositions of several hymenopteran species (Bouzid et al., 2013; Danneels et al., 2015; Liu et al., 2015; Ozbek et al., 2019; Robinson et al., 2018; Tan et al., 2020; Teng et al., 2017; Touchard et al., 2015, 2018; Wang et al., 2016; Yoon et al., 2020). However, the majority of these studies are restricted to a few species of parasitoid wasps, true wasps, ants, honeybees and bumblebees, see e.g. (Cerpes et al., 2021; de Graaf et al., 2009; Schmidt et al., 1986; Walker et al., 2018). Except for species of honeybees (*Apis* spp.) and bumblebees (*Bombus* spp.), bee venoms remain understudied. The long-standing focus on the eusocial honeybees and their close relatives (genera *Apis* and *Bombus*) overlooks that the solitary, wild bees are more speciose and evolutionary older (Branstetter et al., 2017; Peters et al., 2017; Sann et al., 2018). Despite of their tremendous contribution to plant pollination (Klein et al., 2018; Koh et al., 2016), their biology and ecology are still little studied, including their venoms. This taxonomical imbalance is also reflected in the number of curated venom components in the database UniProt (Jungo et al., 2012). From 355 currently manually reviewed venom peptides and proteins, only 12 come from six solitary bee species (Fig. 1).

The few existing studies on solitary bee venom are mostly application-driven and focus on the identification of single compounds in crude venom and their possible bioactivity. A major attention is paid to new antimicrobial peptides from solitary bees (Čujová et al., 2014; Kazuma et al., 2017; Ko et al., 2020; Monincová et al., 2010, 2012, 2014; Raghuraman and Chattopadhyay, 2007; Stöcklin et al., 2010) for which hymenopterans are particular known for (Moreau, 2013; Slaninova et al., 2012). The only proteomic study using whole solitary bee venom is the one of the Japanese carpenter bee *X. appendiculata circumvolans* (Kazuma et al., 2017), however it also focuses on shorter peptides. It may well be that this underrepresentation is linked to often lower specimen abundances and the small sizes of body and venom systems of most solitary bee species, which make them difficult to study. Nevertheless, to understand the origin and evolution of venom in bees, it is important to analyse venoms from older bee lineages and to include more solitary bee species.

**Fig. 2:**
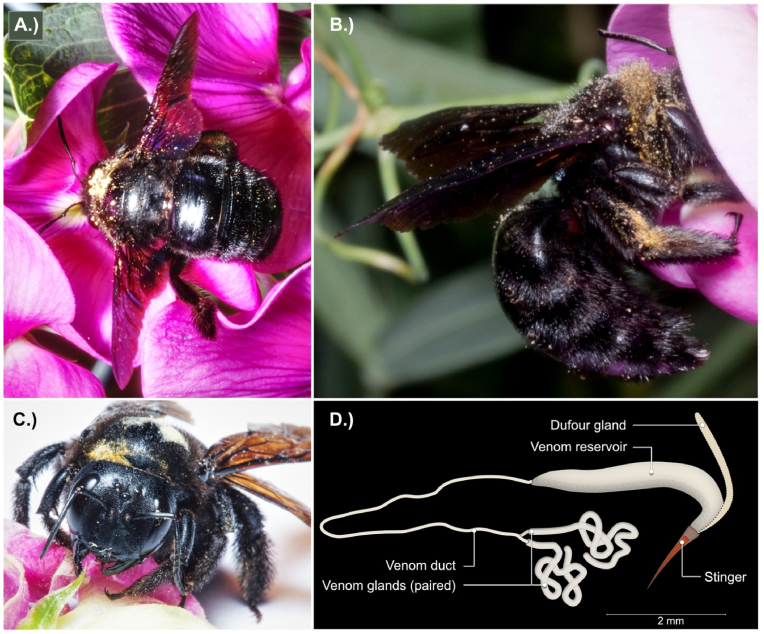
The habitus of *X. violacea.* Shown are individuals collecting nectar while pollinating in A.) and B.). The typical habitats are dry and warm biotopes, such as orchard meadows or allotment gardens with sufficient deadwood in range. These pictures were taken in the collection location of allotment gardens in Wieseck, Giessen, Germany. Despite the species being polylactic and pollinating a variety of plants, *X. violacea* seems to favour lip petals (Lamiaceae) in the collection area and was picked from wild pea plants. In the bottom left picture, C.) the strong mandibles are seen which are used to carve the brood tubes into wood. The venom system composed of venom reservoir (also referred to as poison sac), venom duct and the paired venom glands are illustrated in D.).

The herein studied carpenter bee *Xylocopa violacea* grows up to 2.5 cm making it one of the largest solitary bees in Europe. With its impressive size and dominant blackish colour this solitary bee is often mistaken for a bumblebee or even a beetle. Characteristic nesting behaviour gives the group its name: carpenter bees carve their tubes and breeding chambers into living or dead wood with their large and powerful mandibles (Fig. 2A–C). Interesting in this context is that *X. violacea* appears to expand its range because of the climate change (Banaszak et al., 2019). Probably due to their size and striking habits, the first report on carpenter bee venom was published in 1865, in which the observation was made that its venom could kill a small bird within a few hours (Bert, 1865). And it has long been known that for humans, stings by *Xylocopa* can be very painful (Hardouin, 1948; Hermann and Mullen, 1974). Despite having this potent weapon, carpenter bees are not very aggressive and females only sting as a last resort, especially when protecting the brood. An ironic sidenote here is that the males behaviourally put on quite a show by darting and buzzing very menacingly towards potential aggressors despite actually being stingless and only mimicking a female's stinging attack. The only human fatality reported following envenomation by *Xylocopa* was from Sri Lanka, but even that one case was later attributed to an unfortunate anaphylactic shock (Kularatne et al., 2016).

## 2 Material and methods

### 2.1 Collection of specimens and proteo-transcriptomic samples

Specimens of *X. violacea* (15 individuals) were collected in July and August 2019/2020 in the alluvial area of the River Wieseck in Giessen, Germany, with the permission to BMvR from the HNLUG Giessen (IV.2 R28). From each individual, the whole venom system composed of venom reservoir, venom duct and venom glands (Fig. 2 D) was dissected on ice under sterile conditions. Striking for *X. violacea* is that the venom reservoir is rather large and shows a very long and stretched tube-like shape in contrast to rather small and round or sack-like reservoirs seen in bees and wasps (Fig. 2 D).

The large and long Dufour gland was excluded and preserved for separate analyses. Crude venom was extracted from glands and venom reservoirs by squeezing with forceps in sterile ultrapure water (Thermo Fisher Scientific, Waltham, MA, USA) after prewashing twice to minimize haemolymph contamination. The tissue of the venom system was then preserved in RNAlater (Thermo Fisher Scientific) to generate transcriptomic data. RNA extraction, library preparation and short-read genome sequencing were outsourced to Macrogen (Seoul, Korea). RNA was extracted in Trizol and a low input protocol (Illumina Truseq) was used for library preparation. Libraries were sequenced (150-bp paired-end reads) on an Illumina HiSeq 2500 platform (Macrogen). Complementary data of defensively injected venom was generated by holding individuals (n = 3) between large forceps and inducing them to sting into sterile parafilm-sealed centrifuge tubes filled with ultrapure water. Due to difficult seasonal variations in populations during collection period, these individuals were collected in France, near Montpellier.

### 2.2 Transcriptome assembly, protein prediction and annotation

The venom gland transcriptome was assembled with the Oyster River Pipeline v2.2.6 (MacManes, 2018) which operates multiple assemblers (Trinity, RNASpades with 55 and 75 kmer length and Shannon using a kmer-length of 75) applying standard settings in the provided Docker image (MacManes, 2018). For further details see (Koludarov et al., 2022). The assembly was then processed running Transdecoder (Minimum length ≥20 amino acids) to predict open reading frames (ORFs) for peptides (Haas, Release v5.5.0), and Kallisto v0.46 (Bray et al., 2016) to calculate individual transcript abundance. The longest ORFs were used as local BLAST queries against ToxProt and UniProt (the latter limited to apocritans only) with an e-value cutoff of 1 × 10^−3^. For subsequent venom protein identification, we only include transcripts that were identified in our proteome data of dissected and injected venom (Supplementary Table 1). The transcriptome raw data is accessible in NCBI under the BioProject PRJNA733472 with the SRA entry SRR14690757.

### 2.3 Proteomic analysis of crude venom

Prior to shotgun proteomics, the *X. violacea* venom samples (dissected and injected) were denatured, reduced, and alkylated. Each sample (∼50 μg) was dissolved in 89 μl 100 mM triethylammonium bicarbonate (TEABC), and cysteine residues were reduced by adding 1 μl 1 M DTT (30 min at 60 °C) and modified by adding 10 μl 0.5 M iodoacetamide (incubation for 30 min in the dark). Then 2 μg trypsin (Promega) were added in 100 mM TEABC and incubated overnight at 30 °C. The resulting peptides were purified and concentrated using OMIX Tips C_18_ reversed-phase resin (Agilent Technologies, Santa Clara, CA, USA). The peptides were dehydrated in a vacuum centrifuge and analysed by NanoLC-MS/MS. Samples were resuspended in 20 μl buffer A (0.1% formic acid) and 1 μl was loaded onto an analytical 25 cm reversed-phase column (Acclaim Pepmap 100 C_18_) with a 75 mm inner diameter (Thermo Fisher Scientific) and separated on the Ultimate 3000 RSLC system coupled via a nano-electrospray source to a Q Exactive HF-X mass spectrometer (Thermo Fisher Scientific). Peptides were separated using a 6–40% gradient of buffer B (80% acetonitrile in 0.1% formic acid) over 123 min at a flow rate of 300 nl/min. Using data-dependent acquisition mode, full MS/MS scans (375–1500 *m/z*) were performed in the Orbitrap mass analyser (Thermo Fisher Scientific) with a 60,000 resolution at 200 *m/z*. For the full scans, 3 × 10^6^ ions accumulated within a maximum injection time of 60 ms. The 12 most intense ions with charge states ≥2 were sequentially isolated to a target value of 1 × 10^5^ with a maximum injection time of 45 ms and were fragmented by higher-energy collisional dissociation in the collision cell (normalized collision energy 28%) and detected in the Orbitrap mass analyser at a resolution of 30,000.

All transcriptome assembly-based predicted ORFs were used as specific databases to identify peptides and proteins detected by mass spectrometry for the two dissected and injected venom samples using PEAKS Studio v8.5 (Bioinformatics Solutions, Waterloo, ON, Canada). Carbamidomethylation was set as a fixed modification, and oxidation of methionine as a variable modification, with a maximum of three missed cleavages for trypsin digestion. Parent and fragment mass error tolerances were set at 5 ppm and 0.015 Da, respectively. A false discovery rate (FDR) of 1% and a unique peptide number ≥2 were used to filter out inaccurate proteins. A −10lgP value > 120 was used to estimate whether detected proteins were identified by a sufficient number of reliable peptides. In order to identify more relevant sequences, the Spider algorithm (PEAKS Studio) was used to find additional mutations or to correct sequences. This algorithm corrects the sequences stored in transcriptomic databases with *de novo* sequences based on MS/MS spectra, allowing the detection of post-translational modifications (PTMs) and mutations. The minimum ion intensity for PTMs and mutations was set to 5%, and the ALC score was set to ≥90 for *de novo* sequences, leading to low precursor mass errors. Transcripts supported by proteomic data were manually filtered by excluding non-venom-related proteins and peptides, such as house-keeping and structural genes (Supplementary Table 1). The proteomic raw data is submitted to PRIDE with the dataset identifier PXD029823 for dissected venom proteomic data (first published in (Koludarov et al., 2022)) and PDX030997 for injected venom proteome data.

### 2.4 Phylogenetic analyses of major venom components

Sequences of melittin, apamin and MCDP peptides were aligned with the software Mafft (Katoh and Toh, 2008), applying the algorithm mafft-L-INS-I. The rather short and highly similar sequences of the discussed peptides make the use of tree reconstructions difficult, also because substitution models are difficult to fit. We used therefore as alternative neighbour networks that indicate in contrast to strictly bifurcating trees also ambiguous and competing relations of sequences. The networks were reconstructed using Splitstree 5.3.0 (Huson, 1998; Huson and Bryant, 2006) applying the Protein ML JTT model for underlying distance matrix reconstructions. All other sequences of venom proteins were aligned applying mafft-E-INS-I.

## 3 Results

### 3.1 The general venom profile of *X. violacea*

**Fig. 3:**
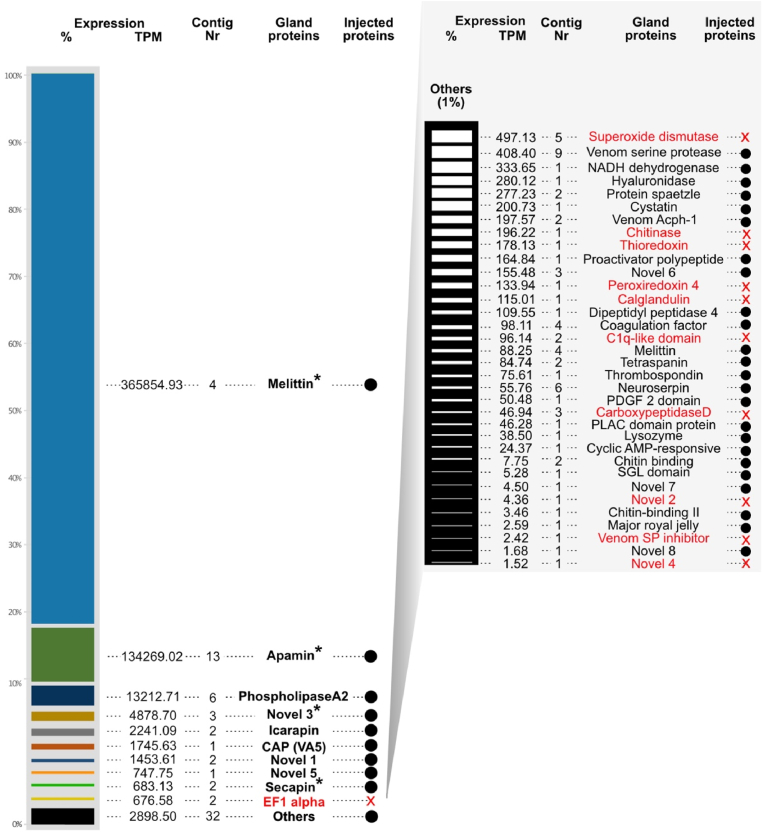
Venom profile of *X. violacea.* For the proteo-transcriptomic data based on dissected venom glands (‘gland’ proteins) only transcripts were considered that were identified in the proteomic data. Expression values are given in TPM (transcripts per million) and are rounded on the second position after the comma. Transcripts that were also identified in crude venom obtained from agitated stings (injected venom) are marked by a black circle, while proteins that were only identified via dissection of glands are marked in red, see also Supplementary Table 1. Asterisks indicate peptides.

In total, we matched 199 transcripts with the peptide fragments from the mass spectrometry data of the dissected and injected venom samples. 43 different venom protein groups had TPM values above 1.5 (Fig. 3, sorted from the highest to lowest expression value). Only five of these are composed of short peptides, while other 38 families represent larger peptides or proteins. Interesting, however, is that four of these peptide groups (melittin, apamin, secapin and novel 4) are among the nine most highly expressed components.

By far the most dominant secreted venom components in the dissected and injected venoms are well-known major honeybee toxins (Pucca et al., 2019; Wehbe et al., 2019). They belong to the two peptide families of melittin (TPM 365773.84, ∼70%) and apamin (TPM 134017.92, ∼25%), and the enzyme family phospholipase A2 (TPM 13212.71, ∼2%), see Fig. 1. Other dominant but far less expressed components are three novel protein families 5, 6, and 12 (TPMs of 4878.70, 747.75 and 1453.61), icarapin (TPM 2241.09), venom allergen 5 (TPM 1745.63) and secapin (TPM 683.13). All other components of the remaining 34 families are far less abundant with TPM values under 500, except for EF1 alpha (TPM 676.58), which was, however, not identified in the injected venom. Eleven families are represented in the venom from the dissected venom glands only and are not identified in the injected venom (See Fig. 3).

### 3.2 The most dominant venom components are melittin-like peptides

**Fig. 4:**
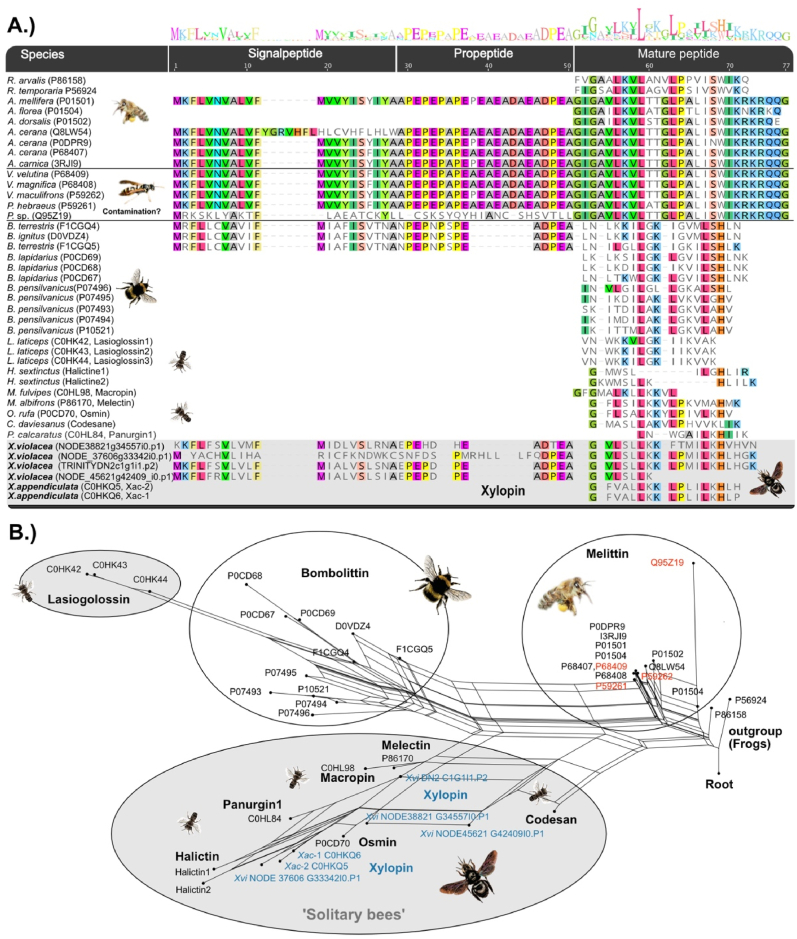
Sequence alignment of available melittin sequences reconstructed with MAFFT-L-INSI (A). Neighbour networks reconstructions based on distance matrices (Protein ML JTT model) are shown for entire sequences; (B). Identifiers in red represent wasp sequences, sequences from *X. violacea* are highlighted in blue. For outgroup rooting we used melittin-like peptides from the European common frog (*Rana temporalis,* P56924*)* and the moor frog *(Rana arvalis*, P86158).

Our data reveals that melittin-like peptides are by far the most expressed venom component. This is not surprising since melittin is known as the most dominant venom component in honeybee venom where it makes 50–60% of dried venom mass (Pucca et al., 2019). The four melittin-like precursor sequences in our transcriptome feature similar signal and propeptide regions compared to known *Apis* and *Bombus* sequences, see Fig. 4. The mature sequences are very similar to two antimicrobial peptides named xylopin (Xac-1, Xac-2) that previously were identified in crude venom from the Japanese carpenter bee *Xylocopa appendiculata crimuvolans* (Kazuma et al., 2017). Because the short sequences in our alignment make it difficult to fit evolutionary substitution models, we used a distance matrix-based neighbour net reconstruction to visualise phylogenetic relations between known melittin and melittin-like sequences. We generated two separate alignments of available melittin-like sequences, one including the complete precursor sequences available for honeybees, bumblebees and our *X. violacea* transcripts (Fig. 4 B), and one that includes the mature sequences only (Supplementary Fig. 1). Both rooted networks show noise in the alignments, and alternative possible connections of sequences indicate either ambiguously aligned positions or conflicting signal (for alignments see Supplementary Files 1 and 2). However, both networks reveal distinct groups of which two are the honeybee melittins and the bumblebee melittin-like sequences. Within solitary bee melittin-like sequences we find a grouping of peptides from the solitary bees *Osmia rufa* (Osmin), *Xylocopa violacea/Xylocopa appendiculata circumvolans* (Xylopin), *Macropis fulvipes* (Macropin), *Melecta albifrons* (Melectin), *Colletes daviesanus* (Codesane), *Panurgus calcaratus* (Panurgin1) and *Halictus sexcinctus* (Halictin) for which a relation to bombolittins or melittins remains ambiguous. The melittin-like peptides from *Lasioglossa laticens* (Lasioglossin) appear to be closer related to bombolittin sequences than to other solitary bee peptides. Our analysis also indicates that the melittin-like sequences published for *Polistes*, *Vespa* and *Vespula* wasp species probably are contaminations or mistaken samples being identical to bee melittin sequences, see Fig. 4.

### 3.3 The second dominant venom component are apamin-like peptides

**Fig. 5:**
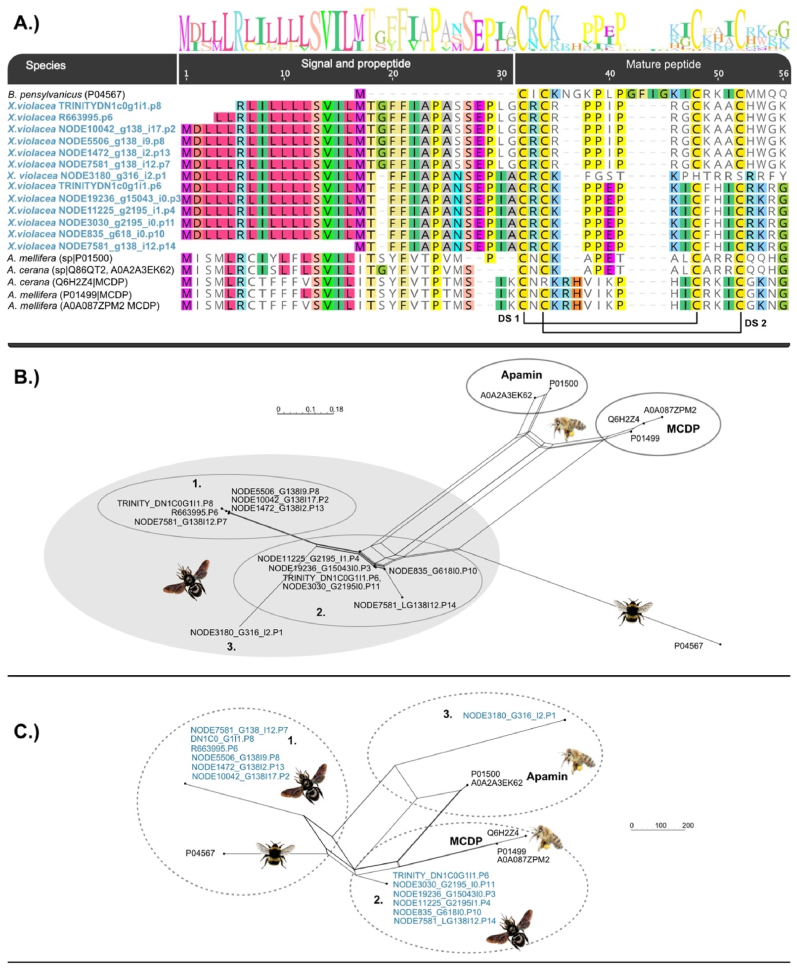
Sequence alignment of available apamin and MCDP sequences reconstructed with MAFFT-L-INSI (A). Neighbour networks reconstructed based on distance matrices (Protein ML JTT model) are shown for entire sequences (B) and mature sequences (C). Sequences from *X. violacea* are highlighted in blue.

The second highly expressed component in *X. violacea* are peptides that are very similar to apamin, a second-highest expressed toxin in honeybee venom (Pucca et al., 2019), and MCDP, which is a peptide that resembles apamin very closely. In our upcoming study (Koludarov et al., 2022) using genomic data we show that apamin and MCDP are not only closely related to each other but also belong to one large peptide family, anthophilin1, which is restricted to bees. Interestingly, the syntenic information of the genomic regions indicate that MCDP is only occurring in *Apis*. Characteristically, apamin and MCDP feature two disulphide bonds that fold the mature peptide by connecting the 1st with the 3rd and the 2nd with the 4th cysteine residue, see Fig. 5 A. The 13 apamin-like transcripts that we identify for *X. violacea*, appear to be more diverse and split into three separate clades with six transcripts for clade one and clade two, which encode the same peptide sequences. The third clade is represented by a single transcript that lacks the 3rd and 4th cysteine but shows an identical signal and propeptide seen in clade two Fig. 5 A.

To investigate the relationships between the apamin clades in *X. violacea* we again used neighbour network reconstructions of the complete and mature transcript sequences including available sequences of apamin and highly similar mast cell degranulating peptides (see alignments given in Supplementary Files 3 and 4). The network based on the full sequence rather separates the species clades (*Apis*, *Bombus* and *Xylocopa*), see Fig. 5 B. It appears that three groups of apamin-like sequences can be recognized.

The network of the mature sequences (Fig. 5 C) also distinguishes three groups of apamin-like sequences in *X. violacea*. Clade two clusters with MCDP from *Apis*, while peptides from clade one are more similar to the MCDP sequence of *Bombus* (which probably is an Apamin). The single transcript in clade three seems more closely related to apamin from *Apis.* However, it becomes apparent that the resolution of the short sequences is too low and ambiguous relationships are apparent that cannot be resolved based on this dataset.

### 3.4 Dominant larger venom peptides and proteins

Several larger proteins were identified in the venom which are, however, far less abundant compared to melittin and apamin (Fig. 3). Phospholipase A2 is with six transcripts (TPM 13212.71) the third most expressed venom component in *X. violacea* (see Supplementary File 5 for its alignment). In honeybee and bumblebee venoms phospholipase A2 is well known as a highly expressed major toxic component that is enhanced in its effect to attack membrane phospholipids by the pore forming melittin (Pucca et al., 2019).

Icarapin is another major allergen known from honeybees (also referred to as Api m 10). So far it has only been described directly from the venom of *Apis* species mostly in the context of bee venom treatment. However, matches from genomic sequence data to other solitary bees, wasps and ants suggest that this protein is also present in other bees and hymenopterans, which is confirmed here for the solitary bee *X. violacea* (See Supplementary File 6 for aligned sequences).

Venom allergens are a large protein family of which representatives occur in diverse venoms of all kinds of venomous animals (CAP superfamily). Venom allergens 5 sequences that we identified in *X. violacea* are similar to variants from wasps and ants, but also to sequences identified in genomic data from *Apis* and *Bombus* venom (See supplementary File 7 for aligned sequences).

Secapin was described in *Apis* and positively tested for various bioactivities, predominantly antimicrobial or antifibrinolytic (Pucca et al., 2019). Only available non-honeybee crude venom evidence comes from ants and suggests a higher similarity between ant and bee secapins. The two transcripts identified in *X. violacea* are more distinct from *Apis* secapin and also have a different mature peptide, with four instead of two Cysteine residues. We have to note that similar to the picture in melittin, the secapin sequences that derive from wasps are close to be identical to bee sequences (mostly 100%), which again indicates a possible contamination or erroneous database upload (See Supplementary File 8).

### 3.5 Novel venom components

Finally, our analysis reveals eight novel venom components for which only vague annotations can be given. For that reason, we refrain here from a more detailed discussion and refer to Table 1 and Supplementary Table 1. Nevertheless, it is striking that the cleaved products from these novel precursor sequences are predominantly peptides, of which several show Cysteine scaffolds (Table 1). This always hints towards a peptide folding based on disulphide bridges which might indicate interesting bioactivities for applied research. Except for number two and four, all novel proteins are identified in the injected venom as well.

**Table 1:** Overview of novel venom components identified in *X. violacea.* See Supplementary Table 1 for further details on annotations. Components that are present in the injected venom are indicated by an asterisk. SP column shows the length of predicted signal peptides in amino acid positions. All sequences are given in Supplementary Files 9 to 16.

Name	Transcript name	Length	SP	Pattern
Novel 1*	R662262.p2	94 aa	15	X (3)-C-X (45)-C-X (5)-C-X (5)-C-X (11)
Novel 1*	NODE17211_g13121i0.p1	83 aa	15	X (3)-C-X (45)-C-X (5)-C-X (5)-C- (truncated)
Novel 2	DN7443_c0g3i1.p4	53 aa	NA	Leucin, Lysn, Isoleucin rich
Novel 3*	NODE12433_g1692_i3.p4	54 aa	26	X (2)-C-X-C-X (2)-PPP-X (2)-P-X (5)-C-X (3)-C-X (4)
Novel 3*	NODE4379_g3275i0.p6	54 aa	26	X (2)-C-X-C-X (2)-PPP-X (2)-P-X (5)-C-X (3)-C-X (4)
Novel 3*	NODE7033_g159i1.p3	54 aa	26	X (2)-C-X-C-X (2)-PPP-X (2)-P-X (5)-C-X (3)-C-X (4)
Novel 4	NODE26737_g22473i0.p1	118 aa	NA	Serine, Isolecuin, Asparagin, Isoleucin rich
Novel 5*	DN2824_c0g1i3.p3	71 aa	42	X (4)-C-X (7)-C-X (8)-C-X (3)-C-X (3)
Novel 6*	R662790.p8	42 aa	NA	Isoleucin, Lysine, Valine, Arginine rich
Novel 6*	DN49_c0g1i1.p7	42 aa	NA	Isoleucin, Lysine, Valine, Arginine rich
Novel 6*	DN49_c0g1i9.p7	42 aa	NA	Isoleucin, Lysine, Valine, Arginine rich
Novel 7*	J678067.p1	445 aa	NA	NA
Novel 8*	DN10104_c0g1i1.p1	165 aa	NA	Contains predicted disorder-regions

## 4 Discussion

### 4.1 *X. violacea* has a complex venom profile with several unique components

The honeybee (*Apis*) is among the very few hymenopteran taxa for which the venom has been known in a great detail for quite a long time (Piek, 1986; Pucca et al., 2019). This predominant focus on the honeybee facilitates even the misleading use of the term ‘bee venom’ by referring only to the honeybee venoms instead of the whole clade Anthophila (bees *sensu lato*). It is known by now that honeybee venom composition and production can alter during the year in worker bees and that queens have a different venom composition (Danneels et al., 2015; Scaccabarozzi et al., 2021). A recent study on eusocial bumblebees (*Bombus*), the sister group to *Apis*, showcases further that venom compositions are dynamic and vary according to the habitat altitude (Barkan et al., 2020). To understand these obviously highly dynamic adaptations in bee venoms and their general mechanisms in more detail a broader understanding of bee venoms *sensu lato* is necessary, as well as work comparing honeybee and bumblebee venoms to other evolutionary older solitary (non-eusocial) species of bees. The herein discussed venom composition of *X. violacea* with 32 proteinaceous venom components identified in its injected venom and 43 components in the dissected venom system illustrates that solitary bees might have a very complex venom composition. The components which are absent in the injected venom are likely chaperone proteins and enzymes (such as dismutase, carboxypeptidase) that facilitate the maturation of the venom components. To better understand the complexity and possible adaptations of envenomation processes and immunology in bees (*sensu lato*), more data on injected venom components (which interact synergistically) are important from different bee lineages. Most injected venom proteins (Fig. 3) we identified in *X. violacea* belong to groups shared with honeybees and bumblebees (Barkan et al., 2017, 2020; Pucca et al., 2019). However, it is striking that novel peptide classes are found in the 9 most highly expressed components of the carpenter bee venom, for which activity and function remain to be studied. The question if these occur also in venoms of other solitary bees or if they are possibly unique to carpenter bees cannot be addressed without further studies of other carpenter bee species' and other solitary bee species' venoms.

### 4.2 Insights into the evolution of bee venom components

**Fig. 6:**
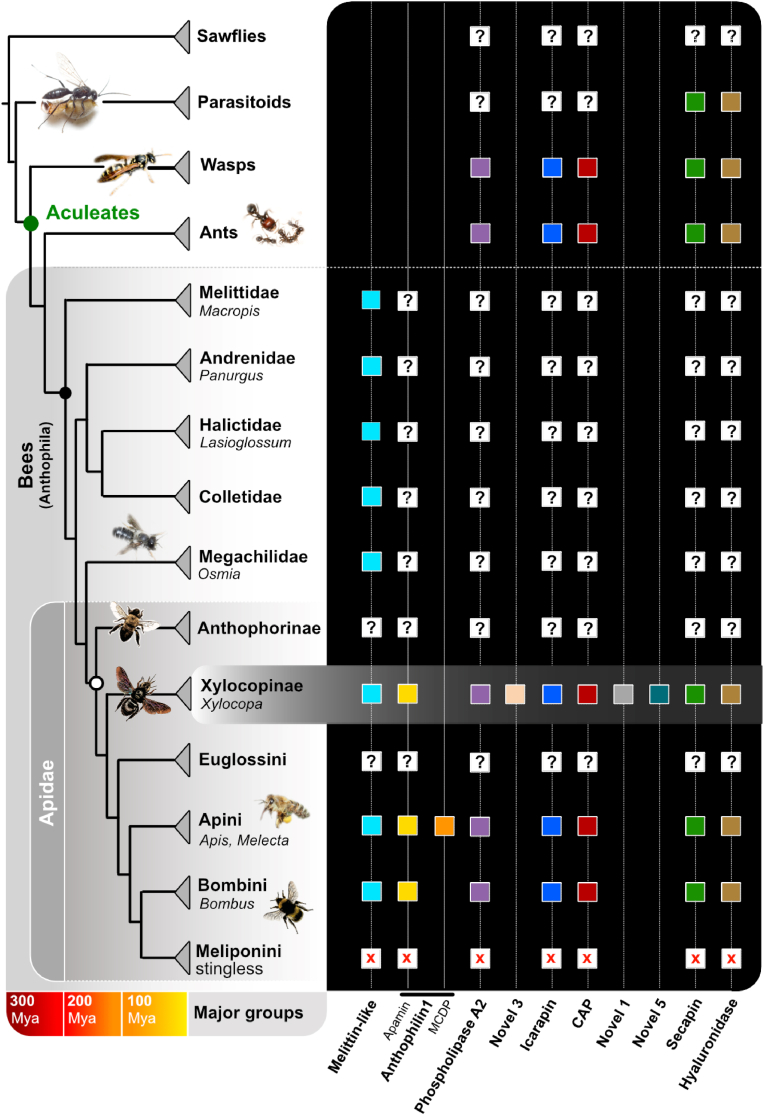
Most expressed venom components of *X. violacea* in the context of bee phylogeny and available proteomic data of bees. Red “X” marks indicate missing or reduced venom system from stingless bees (Meliponini). Phylogeny and divergence times are according to (Peters et al., 2017; Sann et al., 2018).

Melittin that was first described in honeybees represents their most dominant venom component and is probably the singular best studied Hymenopteran toxin (Pucca et al., 2019). Melittin-like variants (Bombolitin) are also expressed in venom of *Bombus* (Argiolas and Pisano, 1985), despite bumblebee venom being dominated by phospholipase A2 in contrast to honeybee venom (Barkan et al., 2017, 2020; Yoon et al., 2020). It was illustrated in recent proteomic studies that most venom components from *Apis* and *Bombus* are generally very similar (Barkan et al., 2017; Van Vaerenbergh et al., 2015), which is probably linked to the close phylogenetic relationship of these two genera (Peters et al., 2017), see also Fig. 6. We show that in *X. violacea* most venom components are also similar to homologs known from *Apis* and that melittin is the most dominant venom component. The network reconstruction (Fig. 4) indicates that most melittin-like peptides from solitary bees form a single clade to the exclusion of their bumblebee and honeybee counterparts. In general, melittins from *Bombus* and *Apis* are more distinct from the ones found in solitary bees and only lasioglossins from *Lasioglossa* bees appear closer related to bombolitins. However, our networks clearly indicate that short but partially more distinct peptides are of limited use for the analysis of phylogenetic relationships due to the higher impact of randomly similar aligned amino acids which subsequently can impede appropriate model fitting. As a consequence, to avoid phylogenetic conclusions based on similarity, alternative methods become important. In our recent genomic analysis, we revealed that melittin genes are found in several available genomes of solitary bee clades including the carpenter bees (Koludarov et al., 2022), see also Fig. 6. Using micro-syntenic patterns of gene regions that flank the exons of melittin in *Apis* we show that this toxin gene is also present in the carpenter bee *C. calcarata* and in the megachilid genus *Osmia*. Searching available wasp genomes, we did not find any matches (neither primary sequence-based nor microsynteny-based) which highly supports our deduction that the sequences published as wasp-melittins must be erroneously assigned (see Fig. 4). From the current data we can deduce that melittin probably evolved already in the earlier bee lineages. However, most of the peptides that we included in our analyses are highly variable and short, which is reflected in the ambiguous relations of sequences that are revealed in the networks (in contrast to strictly bifurcating trees). To prevent false interpretation by noisy alignments and subsequent misled phylogenetic analyses, corresponding genomic data is important that allows to identify the coding genes for these peptides and to reconstruct phylogenies based on the full coding domain sequences and microsyntenic patterns.

Apamin and MCDP are two highly similar toxic peptides originally identified in *Apis* venom that both belong to the recently described toxin family Anthophilin1 (Koludarov et al., 2022). In our network analysis we identified three different forms of apamin-like sequences in *X. violacea*, for which it is hard to derive definite phylogenetic relationships. Interestingly, we showed previously that the gene for MCDP appears unique to *Apis* and was not identified in any other bee clade based on genomic microsynteny analysis. As a consequence, a closer similarity of the apamin-like peptides from *X. violacea* to the *Bombus* sequence (that is from our perspective falsely annotated as MCDP) would reflect instead another distinct apamin variant of which *X. violacea* might have three. To finally test if MCDP is indeed not present in *X. violacea* and supporting its uniqueness to *Apis*, a high-quality genome of *X. violacea* would be needed. Such a genome would be of course important to draw conclusions about the general bee venom evolution as well.

Most other major components in the venom of *X. violacea*, such as Icarapin, Secapin, Phospholipase A2 and Venom allergens (CAP) are also present in other bee venoms and evolved in the early lineage of hymenopterans (Fig. 6). This is also the case for many of the lowly expressed venom components. From a biological perspective the question remains why a predominantly defensive venom of bees (including the herein described venom of *X. violacea*) shows such a complex composition. To address this further, future studies would require more holistic overview of bee venoms from a variety of anthophilan species combining proteo-transcriptomic with genomic data. Likewise it would be important to analyse in a greater detail seasonal or habitual variations of bee venoms, advancing further the existing data (Barkan et al., 2020; Danneels et al., 2015).

### 4.3 Functional and applied aspects of identified venom components

Surprisingly, not only the reason for the complexity of a defensive venom in bees is still not fully understood, but also the biological activities and possible synergistic effects of most of the venom components in bees remain unknown. In general, most studies focus on possible allergenic activity of components in the context of envenomation and possible mitigation if humans are stung, see e.g. (Pucca et al., 2019). Another strong focus is on the effects of short, melittin-like peptides that exhibit higher potential for a variety of translational research objectives, such as promising antimicrobial, anti-tumor and anti-inflammatory activities (Čujová et al., 2014; Monincová et al., 2010, 2010, 2012, 2014, 2010; Slaninova et al., 2012; Stöcklin et al., 2010; Wehbe et al., 2019). Nevertheless, at least for the two major components in honeybee venom, melittin and phospholipase A2, the general mode of action has been described. The various bioactivities of melittin are predominantly based on its pore forming activity which synergistically enhances the effect of phospholipase A2 which disrupts cell membranes by attacking phospholipids that are exposed by melittin, see e.g. (Pucca et al., 2019). One difficulty with melittin is that it is so destructive that its application potential has proved challenging to tame for translational research and to modify and to apply it in a way that its effects are more targeted (Gajski et al., 2016). Given that melittin likely evolved earlier within solitary bees, the phylogenetically older melittin variants discovered in this study from *X. violacea* venom are interesting because their bioactivities could differ or be less strong and more applicable compared to melittin tested from *Apis*.

## 5 Conclusions

In the present study we demonstrate that solitary bee venoms are as complex as their more known honeybee and bumblebee counterparts and deserve more attention from the research community. Despite superficial similarities in terms of protein classes, bee venoms are dynamic systems that continuously tinker with their molecular weaponry. The present study highlights an untapped biodiscovery potential of bee venoms by revealing entirely novel venom peptide classes as well as new forms of well-known medicinally important molecules like melittin and apamin as part of the new anthophilin family. Our study also illustrates the importance of more dedicated bee venom research, in particular bioactivity, genomic and proteo-transcriptomic studies of neglected solitary bee species to better understand the evolution and adaptations of their biochemical defence system.

### Ethical statement

The authors adhered to Elsevier's publishing ethics policy and ethical guidelines for journal publication.

## Author contributions

BMvR, SD, IK: conceptualization, BMvR: Proteo-transcriptomic/Phylogenetic analyses, Visualization, Writing -original draft. SD: Proteomic analyses, methodology. IK, SD, BMvR: Writing – review and editing, methodology, BMvR: Project administration.

## Declaration of competing interest

The authors declare that they have no known competing financial interests or personal relationships that could have appeared to influence the work reported in this paper.

## Figures and Tables

**figs1:**